# Supplementary material for: Are carbon emissions trading and green financial instruments synergistic? -Comprehensive quantitative research based on content analysis
Source: PLoS One. 2024 Mar 7;19(3):e0298601. doi: 10.1371/journal.pone.0298601 (PMC10919601; doi:10.1371/journal.pone.0298601)
Supplement: S1 Data — (ZIP) [file pone.0298601.s001.zip › Data/地级市Z_F工作报告.zip!/║╙▒▒/│╨╡┬╩╨/└┤╫╘╣1⁄2╓┌║┼í╛┬φ┐╦╩2╛▌═°í┐_2015.docx]

各位代表：

现在，我代表承德市人民政府向大会作工作报告，请予审议，并请市政协委员和列席会议的同志提出意见。

一、2014年工作回顾

刚刚过去的2014年，是我市遇到困难较多的一年。面对错综复杂的严峻形势，在中共承德市委的领导下，我们认真贯彻党的十八大和十八届三中、四中全会精神，全面落实省委、省政府各项决策部署，紧紧围绕国际旅游城市建设总目标，按照“发挥优势、高点起步、开放创新、绿色崛起”总要求，牢牢把握京津冀协同发展重大机遇，深入推进“改革创新、生态优先、转型升级、开放发展”四大任务，着力稳增长、调结构、促改革、防风险、惠民生，努力实现市十三届人大二次会议确定的目标任务。

经济实现稳中向好。采取一系列有力措施拉动了即期增长。预计全市生产总值完成1320亿元，增长7.5%。固定资产投资1410亿元，增长17%。全部财政收入196.5亿元，增长2.3%;公共财政预算收入107.6亿元，增长5.2%。社会消费品零售总额443亿元，增长13%。在建亿元以上项目947个，省市重点项目开工率居全省前列。京沈客专、南郊热电项目开工，承德机场、双峰寺水库建设进展顺利，丰宁抽水蓄能电站二期提前启动，张承高速、张唐铁路建设稳步推进。北汽福田一期竣工投产，钒钛基地项目获准开展前期工作，比亚迪新能源汽车项目签约。

转型升级成效显著。坚持在调整中发展、在发展中调整，大力推进“六个千亿元产业”规划实施，三次产业结构不断优化。工业经济提质增效，规上工业增加值完成530亿元，增长7.8%。实施露露、天大钒业、苏垦银河等3000万元以上技改项目260个，技改投资增长13.1%。承德风电百万基地二期工程获批，装机22.9万千瓦的8个分布式光伏项目并网运行，尾矿资源综合利用成为新的经济增长点，新增规上工业企业85家，20家企业荣膺全省“百强民营企业”。农业基础不断巩固，粮食产量143.7万吨，蔬菜、食用菌产量分别达到524万吨和93万吨，新栽和改造经济林105万亩，新增中药材5.7万亩，肉鸡、生猪、肉牛、奶牛规模养殖比重迅速上升。实施千万元以上农业产业化项目163个，新增市级以上龙头企业17家，农业产业化经营率提高到65.5%。第三产业发展提速，鼎盛皇家灯会、山庄冰雪运动、隆化温泉度假成为冬季旅游新亮点，金山岭长城景区入选“全球百佳可持续目的地”，我市被确定为国家旅游标准化示范城市，全年接待游客2930万人次、旅游总收入257亿元，分别增长19%和26%。服务业增加值增速高于生产总值2.5个百分点。

协同发展开局良好。抢抓京津冀协同发展重大机遇，一批核心诉求得到国家和省支持。成功争列国家生态文明先行示范区、国家新能源示范城市和全国首批循环经济示范市，全域纳入国家重点生态功能区转移支付范围。21个重大事项列入京冀“6+1”和津冀“4+1”合作协议，34个重大项目列入省“三个率先突破”工作方案。环首都三县开通与北京城际公交，北师大承德附属学校落成招生，23家医院与京津知名医疗机构建立合作关系。推进与首农、中旅、金隅等企业集团合作，实施项目91个、总投资887亿元。面向“长三角”、“珠三角”扩大开放，省级以上园区达到20个，引进资金518亿元，出口总额突破5亿美元。

改革创新全面提速。破除体制机制障碍，市场活力得到较好释放。市本级取消下放行政审批事项121项，市县全部建立政务服务中心，乡镇便民服务中心覆盖率98%，新增市场主体1.7万户。完成农村宅基地和建设用地确权登记51.9万亩，土地承包经营权确权登记28.8万亩，新增流转面积10.5万亩,供销社综合改革率先在全省开展试点。成功引进中信银行、河北银行和张家口银行，中小企业“新三板”上市实现突破。市内金融机构贷款余额增长17%，外埠金融机构贷款余额增长14%，保险业承保金额达到8000亿元。文化体制改革连续两年荣获全国先进，社会领域各项改革取得新进展。

城乡建设协调推进。坚持以人为本推进新型城镇化。编制完成承德市总体规划纲要、协同发展概念性规划等重点规划，实施重点城建项目423个。改造整治中心城区旧小区16.2万平方米和一批小街小巷，新增各类绿地531公顷，拆除违章建筑4万平方米。新增干线公路通车里程622公里，完成农村公路建设1144公里，开通市区至营子区高速公交和6条城乡公交线路。新建输变电站8个、通讯基站977个。新增集中供热面积452万平方米。县城扩容提质步伐加快，农村面貌改造提升成效明显，316个重点村基础条件得到改善，城镇化率达到44.57%。

生态环境持续改善。坚持把生态作为第一资源、第一竞争力，持之以恒优生态、优环境。坚决向大气污染宣战，拆除燃煤锅炉113座，淘汰黄标车7396辆，整治建筑工地508万平方米，新建绿色矿山、花园式选厂100个，推广生物质炊事采暖炉具5.3万台，市区二级以上天数249天，空气质量保持京津冀前列。节能减排完成省定任务，万元生产总值能耗下降4.9%，实施工业污染治理和减排项目253个，二氧化硫和氮氧化物提前实现“十二五”减排目标。下大力涵养修复生态，植树造林80.4万亩，实施小微型水利工程6200多处，治理水土流失面积555平方公里，国省控断面三类以上水质比例达到84%，被确定为全国“水生态文明建设”试点市。

民生保障不断加强。坚持执政为民，把更多的公共资源用于民生，财政民生类支出达到74.2%。城乡居民收入分别增长10%和11%。新增就业4.4万人，城镇登记失业率3.09%。城乡低保标准分别提高22%和19%，农村五保供养达到省定标准，被确定为“全国社会组织建设创新示范市”和“全国和谐社区建设示范城区”。启动回迁房建设100万平方米，中心城区4030户回迁，开工保障性住房14749套，改造农村危房2万套。14.6万农村贫困人口稳定脱贫，31万人饮水安全问题得到解决。省市级示范幼儿园超过100所，义务教育基本均衡县区达到6个，11个县区全部通过普高验收，河北民族师院实现省部共建。县级公立医院改革实现全覆盖，县乡卫生服务一体化管理走在全省前列，市中医院竣工投用。人口计生工作保持全省先进。丰宁滕氏布糊画和滦平抡花列入国家级非物质文化遗产名录 侯卫东官场笔记。森林草原防火、防汛抗旱和食品药品监管工作力度加大，安全生产形势持续好转。严厉打击各类违法犯罪活动，平安承德建设扎实推进，和谐稳定的局面进一步巩固。

一年来，我们自觉接受人大法律监督、工作监督和政协民主监督，认真办理代表建议和政协提案。深入开展党的群众路线教育实践活动，严格执行中央“八项规定”和“约法三章”，清理清退公务用车137辆，调整清理办公用房3.8万平方米，“三公经费”压减22%。我们加快服务型政府建设，一批热点难点问题得到较好解决。国防动员、双拥共建深入开展，对口援疆、民族宗教、广播电视、外事侨务、防震减灾、邮政通信、妇女儿童、体育、人防、气象、统计、档案、老龄、残疾人等事业取得新成绩。

各位代表!过去的一年，我们遇到的困难前所未有，付出的努力非同寻常，取得的成绩来之不易。这是党中央、国务院亲切关怀和省委、省政府大力支持的结果，是中共承德市委正确领导的结果，是全市人民同心同德、拼搏进取的结果。在此，我代表市人民政府，向全市人民，向人大代表、政协委员，向各民主党派、工商联、无党派人士和人民团体，向驻承人民解放军指战员、武警官兵和政法干警，向一直以来关心和支持承德发展的各界朋友，致以崇高的敬意和衷心的感谢!二、2015年工作总体要求

2015年是全面深化改革的关键之年，是全面推进法治承德建设的开局之年，也是全面完成“十二五”规划的收官之年，做好今年工作意义重大。综合分析，我市既面临较大困难和挑战，又面临重大机遇和有利条件。

从困难和挑战看，国内外发展形势错综复杂，世界经济总体复苏疲弱态势难有明显改观。我国经济社会正经历着“三期叠加”的严峻考验，经济下行压力依然较大。我市正处于爬坡过坎的关键时期，面临着扩大总量与提升质量的双重任务、促进增长与保护生态的双重压力、改善民生与维护稳定的双重责任，各类矛盾相互交织，深层次问题不断显现。一是产业结构矛盾突出。传统产业链条短，抵御市场风险能力弱，新兴产业发展慢，服务业比重低，多元支撑格局尚未形成。二是体制机制不够完善。用市场的办法、创新的思维推动发展的措施不多，特别是多层次运用市场资本能力弱，资金短缺仍是制约发展的主要瓶颈。三是资源环境约束加大。环境资源承载力与加快发展的矛盾更加突出，与其他市相比降低能耗和减少污染排放的空间较小，一定程度上对引资金、上项目提出了更加严格的要求。四是改善民生任务较重。基础设施和公共服务欠账多，社会事业投入不足，城乡居民收入还不高，尚有61万农村贫困人口。五是发展环境尚需优化。项目审批“落地难”的问题还比较突出，一些政府部门和工作人员依法行政意识淡薄，甚至个别领域和地方腐败现象严重。对此，我们一定严肃面对，切实加以解决。

从机遇和条件看，我国经济发展进入新常态，国家将陆续出台稳增长、调结构、促改革、惠民生的一系列利好政策，必将为我们赢得支持、加快发展开辟广阔空间。京津冀协同发展深入推进，我市被定位为京津冀水源涵养功能区，必将为我们更好地发挥比较优势、扩大对外开放、打造新的增长极带来重大机遇。省委、省政府实施创新驱动战略，着力推进服务业大发展快发展，特别是支持我市创建国家级生态文明示范区，必将为我们加快转型升级、绿色崛起提供不竭动力。党的群众路线教育实践活动凝聚了党心民心，众多有识之士涌动着创业激情，一批企业逆势而上、创新突破，进一步积聚起承德发展的正能量。我们必须坚定信心、抢抓机遇、真抓实干，牢牢把握工作的主动权。

今年政府工作的总体要求是：全面贯彻党的十八大、十八届三中四中全会和习近平总书记系列重要讲话精神，认真落实省委、省政府和市委的决策部署，把握京津冀协同发展重大机遇，瞄准国际旅游城市建设总目标，按照“发挥优势、高点起步、开放创新、绿色崛起”总要求，坚持“稳中求进”主基调，主动适应新常态，更加注重改革创新、更加注重开放协同、更加注重转型升级、更加注重生态建设、更加注重改善民生，推进法治承德建设，确保经济持续健康发展和社会和谐稳定。

主要预期目标是：全市生产总值增长7.5%左右，全部财政收入和公共财政预算收入分别增长4%和5%，全社会固定资产投资增长17%，城乡居民收入分别增长9%和9.5%，社会消费品零售总额增长12%。规模以上工业增加值增长7.5%，服务业增加值增长10%。单位GDP能耗下降3%，化学需氧量、二氧化硫、氨氮、氮氧化物削减量以及细颗粒物(PM2.5)年均值达到省政府下达的控制目标。人口自然增长率控制在7.6%。以内。

三、2015年主要工作任务

实现今年经济社会发展目标，必须统筹兼顾、把握关键，以重点突破活跃带动全局，着力抓好八个方面工作：

(一)多措并举力保经济稳定增长。把稳增长作为首要任务，综合施策，确保经济运行处在合理区间。

强化项目投资拉动。把项目投资作为拉动经济增长的关键之举，全力以赴跑前期、抓开工、促落地。力促露露扩能等110个亿元以上产业项目投产达效，确保产业项目投资增长20%以上。双峰寺水库加快建设，承德机场建成通航，张承高速、张唐铁路、遵小铁路竣工通车。推动丰宁抽水蓄能电站二期核准并开工建设，京沈客专、京通铁路电气化改造、南郊热电全面开工，争取钒钛产品深加工、汽车产业基地等项目列入国家“十三五”规划，承秦铁路纳入国家专项规划，力促京锡高速开工，启动中心城区轨道交通可研编制等相关工作，争列省重点项目13个以上，实施市重点项目100个、超亿元项目800个以上，谋划储备亿元以上项目1200个。进一步完善项目工作机制，坚持抓大不放小，既要顶天立地的大项目，也要铺天盖地的小项目，固定资产投资完成1635亿元。

加大企业帮扶力度。搭建服务企业平台，对生产经营困难的工商企业和房地产企业分类施策，在银企对接上要有新举措、新成效，确保企业资金链不断、市场不丢。对列入省“三个一百”企业和工业“50强”企业进行重点扶持，加快打造10个百亿元集团、30个10亿元企业。筛选100家成长性好的中小企业进行重点培育，支持“个转企”、“小升规”、“规上市”，推动小巨人企业向大企业迈进，新增规上企业100家以上。

鼓励支持全民创业。最大程度地激发大众创业、万众创新的热情，加大对高校毕业生、农村转移劳动力等重点创业群体的服务保障，抓好19个省级备案创业辅导基地建设，承德高新区和各县(区)产业聚集区都要建立大学生创业园。加快民营经济发展，鼓励企业家“二次创业”，引导承德在外成功人士回乡创业，让市场主体活力充分涌动，民营经济增加值增长8%以上。

推动县区重点突破。支持滦平、丰宁、兴隆县在园区共建共享、科技成果转化、培育特色产业等方面，加快对接京津，力争在协同发展上率先突破。支持宽城县矿业经济转型提质，支持平泉、隆化、围场县大力发展食用菌、温泉度假、清洁能源等优势产业，打造连接京津与辽蒙、沿海与腹地的经济增长极。支持承德县加快与市区同城化。支持双桥区、双滦区大力发展现代服务业，打造高端文化旅游名片。支持营子区依托现有大企业“有中生新”，推动资源枯竭型城市“老树长新枝”。支持承德高新区创新体制机制，推动重点项目落地。

(二)坚定不移加快产业转型升级。坚持有中生新、无中生有，以增量做大带动存量调优。

改造提升传统产业。利用市场倒逼机制，采用新技术、新模式推动传统产业向中高端迈进，实施亿元以上技改项目100个。做大钒钛制品产业，整合冶金矿山资源，推进黑色冶金采、选、炼一体化发展，加快钒钛基地新厂建设，拉长产业链条，实施氮化钒铁、全钒液流储能电池等一批新材料项目，努力打造中国北方资源综合利用水平最高的钒钛产品和特钢基地。壮大装备制造产业，以输送装备、石油装备、汽车零部件装备、仪器仪表装备为重点，抓好北汽福田汽车零部件、承德阀门工业园等188个项目的实施，推动装备制造业向精品化、系统化、高端化方向发展。做强食品医药产业，推进颈复康中药生产基地、平安高科菊粉、怡达果品等项目建设，研发新产品、打造新品牌、开拓新市场，力争产值超10亿元的企业达到7家，食品医药工业增加值增长8%以上。

培育战略性新兴产业。把发展新兴产业作为赢得未来的强力支撑，推进3个千万千瓦级清洁能源基地建设，新增风光电装机66万千瓦，比亚迪新能源汽车项目开工建设，长河核电项目争取列入国家规划，清洁能源产业增加值占比提高到15%以上。加快大数据核心创业园建设，推进太谷新兴信息技术等项目实施。鼓励支持生物处理、污染防治等节能环保产业发展，开发推广高效节能技术装备及产品，构建以先进技术为支撑的废旧资源回收利用体系。实施尾矿资源综合利用项目100个，加快建设宽城龙城园区微晶产品示范基地和滦平尾矿资源综合利用交易市场，新增企业20家，产值突破150亿元。

提升园区承载能力。实施高新区产业倍增三年推进计划，深化与中关村、上海张江高新区战略合作。加快张百湾、六沟、凤山和各县区省级产业园区基础设施建设，完善配套功能，创新管理体制，建立市县共建项目利益分享机制，避免无序竞争、同质竞争，力争在承接产业项目上实现大的突破。谋划实施一批高层次的国别、省别产业园，推进中欧产业园、韩国食品园、台湾工业园、温州工业园建设，促进企业集中、项目集聚、产业集群发展。

(三)扩大消费促进服务业大发展。发挥消费的基础作用，推动服务业与其他产业深度融合、快速发展。

优先发展文化旅游产业。围绕“两带三环”布局，主动融入京津旅游一体化，大力培育健康养老、温泉冰雪、中药养生、低空飞行、原生态旅游等一批新业态，抓好七家——茅荆坝温泉度假区、唐家湾丹霞地貌旅游区、围场小滦河生态观光等91个重点文化旅游项目，培育文化创新企业30家。推广“唐乡”主题型乡村酒店模式，着力发展景区景点、采摘园、温泉度假区、汽车营地、乡村民宿等旅游设施，打造自驾游最佳目的地。制定出台支持养老养生和体育休闲产业发展的实施意见，推动一批项目开工建设。整顿规范市场秩序，加大宣传推介力度，积极开拓海外市场，旅游接待人数和收入分别增长5%和10%以上。

积极发展现代物流产业。大力发展第三方物流，加快建设交通枢纽型、生产基地型和商贸集散型三类物流聚集区，推进上板城农产品冷链物流产业园建设，新增超亿元物流企业2个，年销售收入超20亿元物流园区达到3个，实现销售收入127亿元。制定发展电子商务“三年行动计划”，推进电子商务与承德传统特色产业融合，创建全国电子商务示范城市 落霞小说。抓好5个大型商业综合体、5条特色街区建设和招商，改造提升农村集贸市场35个，建设小杂粮成品粮油批发市场。

培育发展新兴服务业态。积极发展服务外包业，支持企业承接国内外市场推广、项目研发、人才培养等外包业务。积极发展涉农保险，统筹发展商业养老保险和医疗健康保险。大力实施家政、信息等六大领域消费工程，全面推进“三网融合”，开展物联网应用示范。推动组建护理、旅游职教和汽车服务集团，促进校企合作、产教融合，形成一批综合型、专业型人力资源服务机构。制定科技服务业发展规划，下大力引进一批科技型服务企业，科技服务业实现增加值40亿元。

(四)扶贫攻坚大力发展现代农业。围绕农业强、农村美、农民富，落实惠农政策，加快脱贫致富步伐。

扎实推进精准扶贫。争取国家和省对民族地区、革命老区的政策支持，深入实施燕山——太行山片区和环首扶贫攻坚示范区规划。坚持“一县一业、几县一业”，抓好“林果业、食用菌、中药材、时差菜、马铃薯、养殖业、小杂粮、手工业、乡村游”九大特色扶贫产业，提升兴隆、宽城果品和丰宁、隆化肉奶牛生产加工能力，支持平泉建设全国食用菌行业价格信息形成中心，扩大滦平、隆化中药材和围场马铃薯、时差菜种植规模，建设6大跨县域产业带、9个产业片区和5个家庭手工业生产基地，新增经济林35万亩、食用菌20万吨、中药材10万亩、设施菜4万亩，肉蛋奶产量分别达到55万吨、17万吨和22万吨。集中扶持128个新建档立卡的贫困村，力争每个贫困户掌握1项实用技术、参与1项高效农业增收项目，增加农民产业性、劳务性和财产性收入，确保8万贫困人口脱贫。

加快发展特色农业。立足打造首都绿色有机农产品生产加工基地，深化京承农业合作，培育“绿色、有机、安全”品牌，让更多农产品进京津市场、上首都餐桌。加快木兰皇家等20个现代农业示范园区建设，打造7个农业休闲观光示范带，新增国家农业标准化示范区和知名品牌示范区2个，创建省级以上休闲农业与乡村旅游示范县2个。支持保承中药、神栗、缘天然等龙头企业加快发展，新增省级以上知名品牌5个、驰名著名商标10个，农业产业化经营率达到66%以上。

改善生产生活条件。新建小微型水利工程3000个，新增和改善节水灌溉面积12万亩，治理水土流失500平方公里。以中心城区周边、环首都县、主要交通沿线和旅游区为重点，抓好滦平整体推进县和平泉中心村示范县建设，推进298个村的农村面貌改造提升，在民居改造、新能源开发利用、污水垃圾处理等方面推广新技术、新材料、新装备，推广生物质炊事采暖炉具3万台，改善人居环境，打造美丽乡村。

(五)改革创新增强发展内生动力。把改革创新贯穿于经济社会发展各领域各环节，全面实施创新驱动战略。

推进体制机制创新。紧跟国家和省顶层设计，积极有序推进各项改革。在做好省取消和下放行政审批事项衔接的同时，进一步加大向县区简政放权力度，赋予区一级更大的管理权限，强化事中事后监管，完善“三级平台”、制定“三个清单”，推行“两个代办”，切实提高行政效能。扎实推进农村综合改革，全面推开农村土地承包经营权确权登记。完善农村产权交易平台，积极稳妥推进土地流转，发展多种形式的适度规模经营，培育壮大各类新型农业经营主体。抓好21个国有林场和5个国有牧场改革。推行政府向社会购买服务，规范和支持行业协会、商会等社会服务组织健康发展。

推进财税金融创新。认真贯彻新预算法，深化绩效预算改革，规范政府债务管理。积极推进供销社综合改革，组建农村供销合作银行，打造服务“三农”的新型合作金融平台。大力发展村镇银行，力争两年内实现县域全覆盖。运用“新三板”、中小企业私募债券、投资引导基金和股权投资基金等方式，推动更多企业上市融资。建立健全贷款保证保险制度，严格防范和处置非法集资，严禁各类投资公司、农业合作社、典当行和担保公司超范围经营，防止区域性、系统性金融风险的发生。

推进科技协同创新。借助京津优势，加强产学研科技创新平台建设。建立产业技术联盟和院士工作站，加快建设钒钛新材料、仪器仪表、文化科技融合示范3个国家级基地和中关村国家自主创新承德示范基地，推动尾矿资源综合利用、中国钒钛、航天环境工程、发动机工艺、遥感技术、特种车辆、河北食用菌、围场马铃薯食品等8家研究院建设，培育一批省级工程技术研究中心和产业创新战略联盟，打造北京科技成果转化及科技产业转移承接区。

推进重点产业创新。促进现代制造业与电子信息、现代服务业与传统产业、现代农业与电子商务等产业融合发展，推动承钢公司、可大毛纺等20家企业“两化深度融合”，加快省级产业集群发展。建立市县科技成果交易市场，每个省级园区打造一个科技企业孵化器，争取省级以上科技专项20个，推动创新成果变成实实在在的产业活动，高新技术产业增加值增长12%。设立产业引导基金和科技专项基金，建立科技创业投资、风险投资、科技担保等投融资机构，形成多元支持产业创新的投入保障体系。

推进企业自主创新。实施科技型中小企业创业服务和高新技术企业培育行动，鼓励企业开发具有自主知识产权的产品和技术，培育创新品牌，对新认定国家和省级重点实验室、工程技术研究中心、企业技术中心给予奖励支持，将符合条件的新产品纳入政府采购目录。加强知识产权运用和保护，专利授权达到310件以上，新增高新技术企业5家、培育科技“小巨人”企业5家、科技型中小企业50家。(六)服务首都借力京津协同发展。充分发挥生态优势，扮演好服务与崛起的角色，在协同发展中实现更好作为。

大力改善生态环境。贯彻落实《加快京津冀水源涵养功能区建设的若干意见》，强化标本兼治，源头严防、过程严管、后果严惩，确保生态环境质量在京津冀地区保持领先。以创建国家森林城市为抓手，加强林地草地管护，实施再造3个塞罕坝、京冀水源林、“千湖工程”等重点生态项目，植树造林77万亩。深入开展工业污染治理，坚决淘汰落后产能，综合整治矿山环境，取缔燃煤取暖锅炉40座以上。推广微煤雾化技术，建设洁净煤配送中心，统筹规划建设新能源汽车配套设施。严控扬尘和油气烟气排放，启动餐厨废弃物资源化利用和无害化处理项目，全部淘汰“黄标车”。治理农村面源污染，禁止露天焚烧秸秆。依法严惩环境违法行为，坚决不上影响生态环境的项目，开展环境污染第三方治理，推进污染治理设施建设和运营市场化。建设天蓝水净、风清气爽、独具魅力的生态环境，打造京津高端人群休闲度假的首选地。

实施“三个率先突破”。按照省京津冀协同发展实施方案，推动3个重点领域率先突破。加快交通互联，确定承平高速路线方案，争取津承铁路列入京津冀城际铁路网近期规划开工项目，制定打通与京津22条断头路的时间表、路线图，积极争取北京城郊铁路延伸至承德。开展生态共建，探索产业补偿、项目补偿机制，大力开展滦潮河流域水环境治理，每个县区建设1至2条河流生态治理样板工程。搞好产业对接，深化与京津在新能源、文化旅游、健康养老、教育医疗、商贸物流等领域的合作，谋划实施项目100个以上，完成投资400亿元。

深化对外开放合作。借力国家“一路一带”战略，发挥比较优势，做大做强农产品、中药材等特色产业，鼓励企业积极“走出去”，拓展中亚、欧洲和俄罗斯等国际市场，扩大产品出口份额，新增出口备案企业15家以上，出口总值增长5%以上。抓住南资北移、台资北移、外资北移的机遇，统筹招商资源，完善招商机制，开展面向港澳台、日韩、中东欧的专题招商活动，引进市外资金增长17%以上。

(七)积极稳妥推进城乡一体发展。建设服务首都特色功能城市，促进以城带乡、协调发展。

做优做强中心城市。进一步优化城市空间布局，彰显历史文化名城特色，围绕避暑山庄及外八庙文化遗产保护和利用，严格执行城乡规划管理若干规定，持续推进老城区中疏，限制高度、疏减密度、控制强度。加快工业和商业批发企业外迁，落实老旧住宅小区和“断头路”改造三年计划，年内改造老旧住宅小区33个，整治小街小巷36条，创建精品示范街2条，实施“两河”沿线桥梁、城市出入口、重要节点绿化美化亮化工程，抓好老城区地下管网配套和新城区路网建设，扩大太阳能、风能及保温材料的推广应用。推进智慧城市建设，开展数字城管综合执法，加强小区物业管理，提高城市管理水平。

推动县城扩容提质。坚持产城融合、延续文脉、显山露水，提升县城及小城镇的特色和水平。重点推进126个城镇项目建设，每个县建设1条样板示范街、1个10公顷综合性公园和16公里绿道绿廊，城镇绿色建筑占新建比重达到25%，县城污水和生活垃圾无害化处理率分别达到80%和90%，进一步增强县城的承载带动能力。加快建设御路文化城镇带、环京津城镇带和出海城镇带，抓好平泉县卧龙镇、宽城县峪耳崖镇2个省级扩权强镇试点。适度发展中心村，规划引领分散村庄科学集中。落实户籍制度改革政策，探索城乡居民社保“一卡通”，推进农业转移人口市民化，城镇化率提高1.5个百分点。

完善城乡基础设施。加快城镇基础设施向农村延伸，投资23亿元实施干线公路项目15个，建设农村公路500公里。加快东区、南区供水厂和双峰寺至太平庄污水主管道工程建设，谋划北区、白河供水厂和市区再生水利用、污泥无害化处置项目。新开工电网项目8个，新建通信基站450个。红石砬沟和草芦沟天然气供热工程建成投用，新增集中供热面积200万平方米。

(八)持续用力保障和改善民生。扎实推进以民生为重点的社会建设，让发展成果更多地惠及全市人民。

大力发展社会事业。启动实施第二期学前三年行动计划和市区中小学校布局调整三期规划，扎实推进义务教育均衡发展和职业教育“五大工程”，积极推进高等教育内涵建设，全面提高教育质量。深化县级公立医院改革，抓好重大疾病防控，市儿童医院开工建设，附属医院新城分院投入使用。提升中医药事业发展水平。山庄外庙文化遗产保护工程全部完工。加强公共文化现代服务体系建设，挖掘、保护和传承好非物质文化遗产，市县图书馆、文化馆、博物馆达标。坚持计划生育基本国策，落实计生惠民政策。广泛开展全民健身活动，提高竞技体育发展水平。

健全社会保障体系。继续提高企业退休人员和城乡居民养老金、工伤保险待遇标准，城乡居民养老保险参保率稳定在90%以上，新农合参合率95%以上。实行城镇医疗保险市级统筹，实现市域内异地就医即时结算，城镇基本医疗保险参保率达到96%以上。落实城乡低保、五保供养政策，实施临时救助制度，确保特困群众得到及时救助。凡是上级出台的民生政策，坚决落实到位。

维护社会安全稳定。严格落实安全生产责任制，加强重点行业、重点领域、重点企业监管，打赢森林草原防火、防汛抗旱硬仗。强化公共安全，抓好景区景点、道路交通、消防和人员密集场所安全保障，坚决遏制重特大事故发生。坚持依法治访，有效化解社会矛盾，有效解决拖欠农民工工资问题。加强社会治安综合治理，严厉打击违法犯罪，为全市人民创造安全有序、和谐稳定的社会环境。

各位代表!人民对美好生活的向往就是我们的奋斗目标，我们要尽心竭力为老百姓办实事、办好事。一是积极扩大就业规模。城镇新增就业4万人，农村劳动力转移就业3.8万人，高校毕业生就业率达到85%以上，城镇登记失业率控制在4.5%以内。二是加大住房保障力度。开工建设回迁房110万平方米，新建保障性安居住房6416套，改造农村危房1.6万户。三是加快农村中小学校舍改造。改善义务教育学校办学条件，新建和改造校舍37万平方米。四是全面实现农村饮水安全。年内解决24.8万人饮水安全问题，确保全市所有农村居民都能喝上干净水。五是提升养老助残服务水平。建立健全以经济困难高龄、失能老年人和残疾人为重点的养老助残福利制度，开通“助老一键通”，居家养老服务中心覆盖率达到75%。六是实施新生儿重大疾病免费筛查。对新生儿35种重大疾病开展免费筛查，进行规范化干预、治疗。七是增加全民健身场所。免费开放市区南营子体育场及奥体中心田径场等一批体育场馆，新建和提升山体公园2个。有条件的县区也要向群众开放体育健身场馆。八是改善居民出行条件。大力发展公共交通，加快城乡公交一体化，推广使用清洁能源公交车，规范出租车运营管理，方便居民和游客出行。九是建设便民服务设施。市区新建社区便利店、高新区生鲜直营店100个，配建小区便民市场3处、停车泊位4000个。各县区都要谋划建设一批便民利民服务设施。十是保障饮食用药安全。完善食品药品监控监测体系，隐患排查覆盖面达到100%，4个县通过省食品药品安全示范县验收。群众利益无小事，我们一定始终把人民的冷暖放在心上，以实实在在的行动为全市人民谋福祉!

四、切实加强法治政府建设

坚决维护宪法法律权威，严格依法行政，全面建设法治政府。

(一)坚持依法履职。牢固树立法律底线不能逾越的观念，做到法定职责必须为、法无授权不可为，不行使依法不该行使的权力，不做依法不能干预的事情，认真履行政府职能，坚决纠正不作为、乱作为，坚决克服懒政、怠政，坚决惩处失职、渎职，营造依法办事、公平公正的发展环境。自觉接受人大法律监督和工作监督、政协民主监督、新闻舆论监督及社会监督。

(二)坚持科学决策。把公众参与、专家论证、风险评估、合法性审查、集体讨论决定作为重大行政决策法定程序，建立重大决策终身责任追究制度及责任倒查机制，对违反决策规定、出现重大失误、造成恶劣影响的，严肃追究法律责任。加强对重大行政决策实施情况的评估考核，确保落实到位、执行到位。

(三)坚持权责统一。健全权力运行制约和监督体系，实行分事行权、分岗设权、分级授权，强化内部流程控制，严防权力滥用。加大对财政资金使用、国有资产、政府投资等方面的监管力度，实现重点领域监督全覆盖。加强对环境保护、征地拆迁、城市管理等重点领域的执法监督，严格公正文明执法，切实维护人民群众切身利益。

(四)坚持实干为民。既严以修身、严以用权、严以律己，又谋事要实、创业要实、做人要实，积极主动作为，勇于担当负责，对重大专项、重点项目和民生实事，久久为功常抓不懈，像锤子钉钉子一样，掷地有声、兑现承诺、严明奖惩，以实干惠民生、以实干促发展。

(五)坚持廉洁从政。坚决落实中央“八项规定”和“约法三章”，严格办公用房、会议、接待、出差等管理，推进机关公务用车制度改革，确保“三公经费”只减不增。认真落实党风廉政建设“一岗双责”，一手抓发展、一手抓廉政，守纪律、讲规矩，始终做到心中有党、心中有民、心中有责、心中有戒。

各位代表，新常态迎来新起点，新常态开启新征程。让我们更加紧密地团结在以习近平同志为总书记的党中央周围，在省委、省政府和中共承德市委的正确领导下，紧紧依靠全市人民，凝心聚力，真抓实干，锐意进取，为全面建成小康社会、建设美丽幸福承德而努力奋斗!
